# Thyroid Hemiagenesis: Narrative Review and Clinical Implications

**DOI:** 10.7759/cureus.22401

**Published:** 2022-02-20

**Authors:** Omotara Kafayat Lesi, Ankur Thapar, Nikhil Nanjappa Ballanamada Appaiah, Muhammad Rafaih Iqbal, Shashi Kumar, Dale Maharaj, Abdalla Saad Abdalla Al-Zawi, Shiva Dindyal

**Affiliations:** 1 General and Colorectal Surgery, Basildon and Thurrock University Hospitals, Mid and South Essex NHS Foundation Trust, Essex, GBR; 2 Vascular Surgery, Basildon and Thurrock University Hospitals, Mid and South Essex NHS Foundation Trust, Essex, GBR; 3 Colorectal Surgery, Basildon and Thurrock University Hospitals, Mid and South Essex NHS Foundation Trust, Essex, GBR; 4 Vascular Surgery, Caribbean Vascular & Vein Clinic, Port-of-Spain, TTO; 5 General and Breast Surgery, Mid and South Essex University Hospital Group, Basildon, GBR; 6 General and Breast Surgery, Basildon and Thurrock University Hospitals, Mid and South Essex NHS Foundation Trust, Essex, GBR; 7 General and Breast Surgery, Anglia Ruskin University, Chelmsford, GBR

**Keywords:** thyroid dysgenesis, one lobe thyroid, thyroid cancers, thyroid hemiagenesis, thyroid aplasia

## Abstract

Thyroid Hemiagenesis (THA) is an uncommon, congenital anomaly defined by the absence of one thyroid lobe with or without the isthmus. Reports suggest it may be found more often in regions endemic for hypothyroidism. Genetic abnormalities are thought to have a role based on findings in monozygotic twins. Most cases are sporadic, however familiar clusters have also been documented. It is found more frequently in females. A majority of patients report no symptoms and THA is found incidentally during investigations or intraoperatively. THA is usually associated with normal thyroid function, but it can present with thyroid hypofunction.

Since a majority of patients are asymptomatic, there are no specific recommendations for management. Ultrasound imaging and thyroid scintigraphy using technetium or iodine are useful in diagnosis. Its clinical importance occurs when the remnant thyroid lobe requires excision leading to the lifelong requirement for thyroxine supplementation.

Published English literature (Medline, PubMed, and Embase databases) was searched. Medical subject headings (MeSH) terms used were “thyroid hemiagenesis,” “one thyroid lobe,” and “thyroid aplasia”. Case reports, case series, and original articles were selected to provide a framework for this review.

Articles reviewed were published in the past 20 years. The association of THA with thyroid cancer was explored. In this group, the F:M ratio was 3.25:1. Left THA constituted 53% of cases, right THA in 29.4%, and isthmus absence in 17.6% of cases. Also, the authors investigated the link between THA and hyperparathyroidism, both left and right THA are seen in an equal number of cases in the hyperparathyroidism subgroup. In patients with THA and Grave’s disease, left THA was seen in a majority of cases (86.7%), while an equal number of left and right THA was observed in patients with Hashimoto’s thyroiditis. In addition, congenital abnormalities associated with THA were observed, the left THA was seen in 60% and right THA in 40% of cases of this subgroup.

The summative review provided a detailed insight into the epidemiology, aetiopathogenesis, genetics, symptomatology, diagnosis, and treatment for THA by combining findings and results from almost a hundred research papers from around the world. THA remains a poorly understood, often incidentally detected, abnormality in euthyroid patients undergoing investigations and treatment for other thyroid disorders.

## Introduction and background

**Figure 1:**
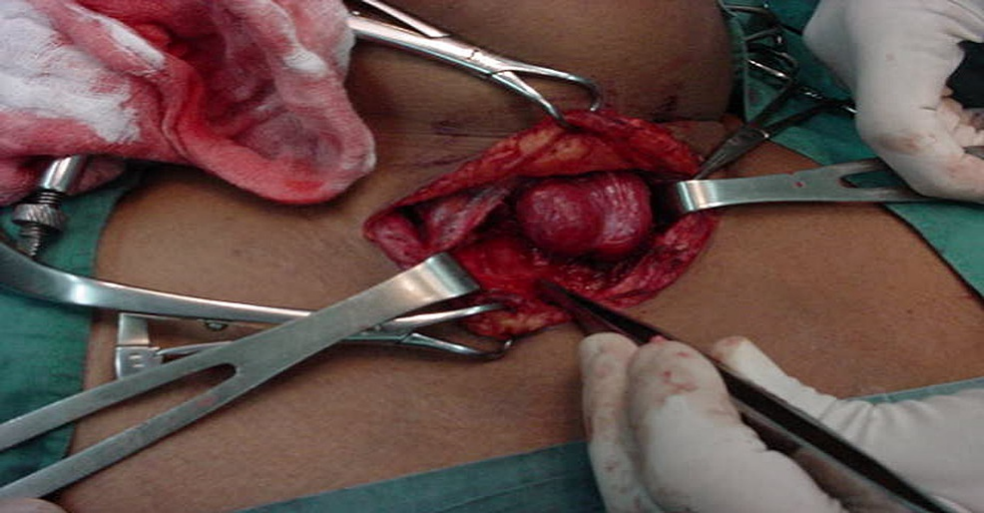
Patient with thyroid hemiagenesis undergoing surgery This image is a property of the Department of Surgery, University of the West Indies, General Hospital, Port-of-Spain, Trinidad, West Indies

Thyroid Hemiagenesis (THA) is an uncommon, congenital anomaly defined by the absence of one thyroid lobe with or without the isthmus [1]. The origin of thyroid gland development starts from the medial thyroid anlage derived from the primitive pharynx while the lateral thyroid anlage is derived from neural multipotent cells. Anomalies in their development can lead to abnormalities in the structure or function of which THA is a part [2]. Genetic abnormalities may have a role to play in the etiology of THA as reported in monozygotic twins [3]. Different studies have shown that in 80% of cases, it affects the left lobe with an L:R ratio of 4:1. The absence of the isthmus is seen in 50% of cases with hemiagenesis of the left lobe while the absence of the right lobe is seen mostly with agenesis of the isthmus [4]. Frequently, THA is an incidental phenomenon as the majority of patients have normal thyroid function. They are discovered during surgery (Figure 1) or diagnostic imaging for other head and neck pathologies [1]. The true prevalence of this anomaly is therefore uncertain because most patients remain asymptomatic and therefore presumably undiagnosed [5]. There is no specific recommendation for management, especially in asymptomatic cases and its clinical importance occurs when the remnant thyroid lobe requires excision leading to the lifelong requirement for thyroxine supplementation [4,6].

## Review

Epidemiology

Handfield-Jones published the first case of THA in the “Cyclopaedia of Anatomy and Physiology" by Robert B. Todd in 1852 [7], also Luschka reported about the pathology in 1876 followed by Ehlers in 1886 [8]. The true prevalence is unknown but is estimated to be between 0.02-0.2% [9], and slightly increased prevalence was reported in an endemic area where there was a high incidence of goiter and thyroid nodules [10]. Children with congenital hypothyroidism have been reported to have a higher prevalence up to 3.7% [11,12]. A study of asymptomatic school children in Northern Poland between the ages of 7-15 years revealed a prevalence of 0.05%. This prevalence was also similar to the study of 11 to 14-year-old schoolchildren in Sicily which revealed a prevalence of 0.05% [5], while Shabana et al. in Belgium revealed a prevalence of 0.2% in asymptomatic school children [13]. Another paper by Gursoy et al. revealed a prevalence of 0.25% in patients with various thyroid disorders [14].

THA is more common in females, however, this could reflect that thyroid diseases are more frequent in females [13,15,16]. A large cohort study of 40 patients by Ruchala et al. revealed a considerable prevalence of women with a Female:Male ratio of 7:1 [17]. A study by Mikosch et al. also revealed a prevalence of 4.3:1 in the 16 patients with THA out of 71,500 patients who had thyroid investigations in 9 years [16].

The most common feature is the absence of the left lobe (missing in 80% of patients) [18]. This set of patients has a greater incidence of associated functional, morphological and autoimmune thyroid disorders such as Hashimoto’s thyroiditis, Graves’ disease in addition to simple and nodular toxic goiter [10]. The presence or absence of the isthmus is not fixed as it is seen in 50% of cases, with a distinctive hockey-stick sign on scintigraphy [19]. A study by Suzuki et al. in Fukushima, Japan to determine the prevalence of THA revealed a statistically significant presence of left hemiagenesis in 55 patients compared to 12 patients with right THA [20].

Embryology of thyroid gland 

The thyroid gland development spans from the third week of gestation to the eleventh week of gestation [21]. It arises from a medial anlage which is larger and a paired smaller lateral anlage [22]. The origin of the median anlage is marked by a permanent pit at the apex of the sulcus terminalis on the dorsum of the tongue known as the foramen cecum. This medial primordium starts from the 3rd week as a proliferation/thickening of the endodermal epithelial cells in the floor of the pharynx inferior to the tuberculum impar (ventral pharyngeal wall) at the border of the first and second pharyngeal pouches. It appears as a duct-like invagination of the endoderm in the floor of the pharynx. This midline structure undergoes numerous transformations like enlargement, bifurcation, lobulations, and detachment from the pharynx [21]. The thyroid gland is initially spherical and then assumes a more bilobed structure as it enlarges [23]. Also, the structure which is initially hollow later solidifies forming follicular elements of the thyroid gland. Division of the gland into lateral lobes, if not present from the beginning, takes place so early that it is impossible to establish whether the thyroid gland arises as a single unit or as a paired organ [24].

During its migration, it descends in front of the hyoid bone and laryngeal cartilages and then settles in its final position anterior to the trachea by the end of the 7th week of gestation [21]. The gland is connected to the foramen cecum by the thyroglossal duct during the migration. The duct later disappears and may remain as a strip of fibrous tissue. The two lobes are located on either side of the midline and connected via an isthmus [24].

The lateral thyroid primordia arise from the ventral part of the 4th and 5th pharyngeal pouches in the ultimobranchial bodies and become attached to the posterior part of the thyroid during the 5th week. It provides 30% of the weight of the thyroid gland [22]. The lateral primordia originate from the neural crest cells (ultimobranchial bodies) and provide the parafollicular C cells which produce calcitonin. At the end of the 7th week, the thyroid gland has a median isthmus and two lateral lobes.

The thyroid follicular cells develop from the median thyroid anlage, begin to appear by the 8th week of gestation and most are formed by the 16th week of gestation. By the end of the 12th week, follicles containing colloid become apparent and begin to incorporate radioactive iodine. Thyroid hormone is produced and secreted into the circulatory system as early as the 10th to 12th week of intra-uterine life [21]. 

Congenital anomalies during descent (i.e. the presence of thyroglossal cysts) are more common compared to the absence of either lobe which is quite rare [25]. The cause of THA is not known and possible theories include failure of descent, defects in lobulation, or genetic aberrations [4].

Molecular patterns for THA

Transcription factors such as *NKX2-1,* *PAX8*, *FOXE1*, *NKX2-5*, *TSHR* have been shown to contribute to the increase and movement of thyroid precursor cells during embryogenesis but mutation in the genes having a role in thyroid morphogenesis has only been reported in a few patients [26]. Most cases are sporadic, however, familiar clusters have also been documented [27].

The *FOXE1* gene is seen on the long arm of chromosome 9 at position 22. It is expressed in the thyroid gland, tongue, palate, choane, and hair follicles. It acts as a thyroid transcription factor and plays a critical function in the development of the thyroid gland. Moreover, it controls the transcription of thyroglobulin and thyroid peroxidase genes. Within the *FOXE1* gene is a coding sequence of polyalanine tract (FOXE1-polyAla) of variable length ranging from 11 to 19 alanines. Szczepanek et al. did a study in 2011 of 40 patients with THA which included 6 familial cases and a control group of 89 patients with normal thyroid glands. The study revealed a short variant of FOXE1-polyAla containing 12 alanines seen in five control patients but was not found in THA patients. The incidence of longer variants >16 codons of FOXE1-polyAla was seen to be significantly higher in patients with the familial form of THA when compared to those with sporadic or the control patients. They concluded that FOXE1-polyAla tract expansion may contribute to the molecular background of familial but not sporadic forms of THA [27].

There has been documentation of heterozygous mutation in Paired box gene 8 (*PAX 8* gene) as a cause. *PAX 8* gene is located on the long arm of chromosomes 12 to 14. It is a part of the paired box family of transcription factors involved in controlling the optimal development of the thyroid gland, Mullerian tracts as well as upper urinary tracts. Macchia et al. reported mutations in *PAX8* with a reduction in the DNA-binding activity of the gene in two sporadic cases and one familial case of thyroid dysgenesis. The first sporadic patient had thyroid ectopy with reduced thyroid gland while the second sporadic case had thyroid hypoplasia. The familial cases had thyroid hypoplasia as well. The study revealed a 100% concordance between *PAX8* mutations and thyroid dysgenesis suggesting that *PAX8* gene plays an important role in the proliferation or survival of differentiated thyroid cell populations [28]. Mutations of *PAX8* gene was also reported in four generations of Jewish-Hungarian family with varying thyroid abnormalities including THA [29].

Szczepanek-Parulska et al., 2021, showed an association of THA phenotype and the presence of compound heterozygous mutations of *GLI3* gene in two siblings with left THA [30]. The *GLI3 *gene is located on the 7p14.1 chromosome. It encodes a cytoplasmic protein that plays a vital part in the development which activates patched Drosophila homolog gene expression. This gene is expressed in various organs including ovaries, placenta, endometrium, and the thyroid gland. The GLI3 protein is a transcription factor and negative regulator of sonic hedgehog (shh) signaling, governing the symmetric lobulation of the thyroid gland. Both sisters had compound heterozygous mutations in the *GLI3* gene affecting exon 14 and 15. These mutations were also present in the daughters of the affected patients but all examined offspring had normal ultrasounds of bi-lobed thyroid gland thus inferring the presence of an autosomal recessive transmission in the *GLI3* gene [30].

THA has been shown to not only be involved with mutations in genes directly involved in thyroid development but also mutations in conservative proteasome genes. In 2017, Budny et al’s study showed 31 of 34 sporadic patients diagnosed with THA and one three-generation family revealed the presence of four recurrent defects (three deletions and one duplication) affecting proteasome genes *PSMA1*, *PSMA3*, and *PSMD3* as well as a splice site mutation in a proteasome gene PSMD2 [31]. Dysfunction of the proteasomal-ubiquitin system has been researched in cardiac diseases and it's known that the development of the thyroid gland is indispensably linked to the cardiovascular system due to the proximity of the thyroid bud to the cardiogenic mesoderm and supported by the presence of heart defects in patients with congenital hypothyroidism [32,33].

Diagnosis/investigation of choice

Ultrasound scan is the first imaging modality of choice as it is sensitive in detecting the absence of the lobe as well as any structural changes to the remaining lobes [11,16,34-36]. It is also widely available, cheap with no risk of radiation to patients. However, it is operator-dependent. Mikosch et al. studied the use of ultrasonography as it was the key investigative tool in diagnosing 16 patients with THA out of 71,500 patients in 9 years [16].

Following ultrasound, thyroid scintigraphy using technetium or iodine detects the functional anatomy of the thyroid gland with the added advantage of being able to detect the presence of hyperactivity and increased diffuse uptake in the glands [36]. Furthermore, scintigraphy can be used to detect ectopic thyroid tissue as well as diagnose thyroid pathologies in the remaining lobe associated with hyperthyroidism or a nodule suspicious of cancer [3]. The disadvantage of scintigraphy is due to artifacts related to the inability to view a thyroid lobe due to cancer, a contralateral autonomous solitary thyroid nodule suppressing normal tissue function, inflammatory and infiltrative pathologies of the thyroid gland, also the use of computed tomography scans have been helpful in further establishing the diagnosis.

Hormonal status with THA

THA is usually associated with normal thyroid function and clinically euthyroid patients with normal levels of thyroxine(T4), triiodothyronine(T3), and thyroid-stimulating hormones (TSH). However, some studies have shown deranged thyroid function tests in patients with THA. Increased TSH levels serve as a growth stimulus for the remaining thyroid lobe leading to hypertrophy i.e., either a diffuse or nodular goitre with an increase in the probability of becoming cancerous [37]. Ruchala et al.'s cohort study of 40 patients with THA reported having higher TSH and FT3 levels when compared to people with the presence of both thyroid lobes. This could be as a result of enhanced peripheral conversion of T4 to T3 or stimulated thyroidal T3 secretion due to elevated TSH in response to thyroid hormones insufficiency. Also, the elevated TSH could result in constant thyroid overstimulation and hypertrophy of the remaining parenchyma lobe [17]. Maiorana et al.'s study of school children in Sicily also revealed similar findings with significantly increased levels of TSH and FT3 in those with THA [5].

Szczepanek-Parulska et al. in 2016, performed a large cohort study of sixty-five patients with THA and revealed a high prevalence of elevated thyroid auto-antibodies with patients having more clinical manifestation of thyroid autoimmune pathology [38]. This could be attributed to a hypothesis in which excessive stimulation of TSH receptors may lead to the “leak” of some thyroid autoantigens such as thyroglobulin or thyroid peroxidase into the circulation leading to an autoimmune response and resultant autoantibody thyroid development [39].

Gurleyik and Gurleyik’s study, 2015, published two case reports of patients with left THA and features of thyrotoxicosis with both cases being from a region of endemic goiter with a history of decreased consumption of iodine in diet [36]. Both patients had suppressed TSH levels with increased free thyroxine (T4) and free triiodothyronine (T3) levels with the second patient having raised antithyroid peroxidase and thyrotropin receptor antibodies with a diagnosis of toxic multinodular goitre and Graves’s disease respectively. Both were initially treated with anti-thyroid medications before having thyroidectomies. Kocakusak et al. in 2004 described a female patient with left THA and absent isthmus with features of hyperthyroidism treated with anti-thyroid medications and surgery [40]. Mortimer et al. in 1981 described four patients with thyrotoxicosis with associated THA. The presence of THA also was identified during thyroid scintigraphy and confirmed during surgical exploration or post-mortem examination [41].

Co-existing thyroidal and extra-thyroidal pathologies

THA can be found in association with clinical pathologies such as nodules, de Quervain thyroiditis, hyperthyroidism [36], thyroid adenomas, Graves’disease [42], or Hashimoto’s thyroiditis [43]; furthermore, THA can interestingly be associated with hyperparathyroidism. Anatomic abnormalities with THA include the absence of an isthmus, the presence of a thyroglossal cyst, a sublingual ectopic thyroid, cervical thymic cysts [44], absence of the thyroid superior and inferior thyroid vessels, and superior or recurrent laryngeal nerves ipsilateral to the missing lobe as well as the loss of the parathyroid gland.

The recent reports showed that thyroid cancer is the fifth most common malignancy diagnosed in women after breast, colorectal, lung, and uterine cancers [45-47] . Studies have shown the association of THA with thyroid cancers. There were twenty-three cases reviewed in this paper (Table 1). The studies were done between 1970 and 2021. The F:M ratio was 4.75:1 with an age range between 14-74 years. Left THA comprised 52% of cases seen, right THA was seen in 34.8% of cases, and absence of the isthmus was seen in 13% of the cases. In twenty patients with true THA, the isthmus was present in nine patients, absent in four patients, and not documented in seven patients. Papillary thyroid cancer was the most common cancer seen as it was documented in 91% of patients. This is also the most common type of thyroid cancer worldwide.

**Table 1:** Summary of case reports of THA in association with thyroid cancer PTC- Papillary Thyroid Cancer; FTC-Follicular Thyroid Cancer; MTC-Medullary Thyroid Cancer; PTMC: Papillary Thyroid Microcarcinoma; PDC: Poorly Differentiated Carcinoma; FV: Follicular variant; ND-Not detected. *Adjacent to the absent left tissue; **Poorly differentiated carcinoma *** Oncocytic variant of papillary thyroid cancer (OVPTC) Huang et al., 2002  [48]; Pizzini et al., 2005 [49]; Ammaturo et al., 2007 [50]; Canani et al., 2008 [51]; Lee et al., 2008 [52]; Karatağ et al., 2013 [53]; Vayisoglu et al., 2013 [54]; Wang et al., 2014 [55]; Campenni et al., 2015 [26]; Sakorafas et al., 2015 [56]; Rajbhandari et al., 2016 [57]; Sato et al., 2017 [9]; Ugur et al., 2019 [58]; Gandla et al., 2020 [59]; Alqahtani et al., 2021 [60]

Study	Patients (n)	Gender	Age	Thyroid Hemiagenesis	Isthmus	Nodule Tomography	Thyroid Tumour
Huang et al., 2002	1	Female	47	Right	Present	Left lobe	PTC
Pizzini et al., 2005	1	Male	54	Left	Present	Right lobe	PTC
Ammaturo et al., 2007	1	Female	39	Left	ND	Right lobe	PTC
Canani et al., 2008	1	Female	35	Right	ND	Thyoglossal duct	PTC
Lee et al., 2008	1	Female	69	Left	ND	Right lobe	PTC
Karatağ et al., 2013	1	Female	59	Left	Present	Right lobe	PTC
Vayisoglu et al., 2013	1	Female	43	Isthmus	-	Right lobe	PTC
Wang J et al., 2014	2	Females	49(60)	Right(Left)	-	Left lobe(Right lobe)	MTC (PTC)
Campenni et al., 2015	1	Male	36	Left	ND	Right lobe	PTC
Sakorafas et al., 2015	1	Female	47	Left	ND	Right lobe	PTC
Rajbhandari et al., 2016	1	Male	28	Isthmus	-	Right lobe	PTC
Sato et al., 2017	1	Female	64	Left	ND	Absent tissue*	PTC +PDC
Ugur et al., 2019	1	Female	54	Isthmus	-	Both lobes	PTC
Gandla et al., 2020	1	Female	20	Right	Present	Left lobe	FV of PTC
Alqahtani et al., 2021	2	Female (Male)	36(40)	Right(Left)	Present	Left lobe(Right lobe)	PTMC (PTC)

The first article describing the association of THA with parathyroid adenoma was by Maganini and Narendran in 1977 [61]. This was seen in a 37-year-old man with left THA and left inferior parathyroid adenoma. A total of 11 cases were reviewed in this current study (Table 2). In six of the 11 reviews, the parathyroid adenoma was present on the ipsilateral side of the THA (five on the left and one on the right). Four of the parathyroid adenomas were located contralateral to the THA while the remaining study had the adenomas located in both left and right parathyroid glands. Of the six ipsilateral cases, three of the adenomas developed from the inferior gland, two from the superior gland while the last one was from both superior and inferior glands.

**Table 2:** Summary of case reports of THA with primary hyperparathyroidism ND: Not detected. Sakurai et al., 2007 [62]; Mydlarz et al., 2010 [63]; Isreb et al., 2010 [64]; Kroeker et al., 2011 [65]; Oruci et al., 2012 [66]; Ferrari et al., 2014 [2]; Eroglu et al., 2015 [67]; Alqahtani et al., 2021 [60]

Study	Gender	Age	Thyroid Hemiagenesis	Site of PA	THA with PA	Size of PA after surgery	Isthmus
Sakurai et al., 2007	Male	42	Right	Left inferior	Contralateral	15x10x10mm 600mg	Present
Mydlarz et al., 2010	Female	55	Left	Left upper and inferior	Ipsilateral	2.2 and 2.4cm	ND
Isreb et al., 2010	Female	75	Left and Isthmus	Left inferior	Ipsilateral	ND	Absent
Kroeker et al., 2011	Male	41	Left	Left inferior	Ipsilateral	1.308gms	ND
Oruci et al., 2012	Female	66	Right	Left inferior, right upper	Bilateral	8x6mm, 15x8mm	Present
Ferrari et al., 2014	Female	15	Left	Right Inferior	Contra lateral	11x5x4mm	Present
Eroglu et al., 2015	Female	27	Right	Right	Ipsilateral	20x11mm	ND
Alqahtani et al., 2021	Female	36	Right	Left inferior	Contralateral	3.5x2.5x1.5cm	Present

The occurrence of Graves’ disease (GD) with THA is uncommon and Table 3 shows a list of the majority of published case reports to date. The clinical features seen with GD are similar to other types of thyrotoxicosis but there is the presence of extrathyroidal features unique to GD including orbital disease, skin and nail changes with about 30-50% of patients having obvious orbitopathy. In the list of case reports, a case of T3 thyrotoxicosis associated with THA was reported in 1982 [68]. In following years, two cases of THA developing GD after hypothyroidism [69,70], one familial form of thyroid dysgenesis [71], one nodular variant GD developing spontaneous hypothyroidism after following medical treatment [72], one case of Down syndrome developing GD following hypothyroidism [73], one multinodular goiter [74] and one hypercalcemia associated with GD [75] were seen. The first reported case of THA and GD in the UK was by Faulkner et al. in 2019. The patient was a 31-year-old female with left THA and GD who subsequently had right hemithyroidectomy and isthmusectomy [25].

**Table 3:** Summary of case reports of THA with Graves Disease THA: Thyroid hemiagenesis. Veliz and Pineda, 2000 [78]; Hervas Benito et al., 2001  [79]; Zangeneh et al., 2001 [80]; Lee et al., 2003 [52]; Ozgen et al., 2004 [81]; Baldini et al., 2005 [72]; Ruchala et al., 2008 [70]; Nebesio and Eugster, 2009 [73]; Kebapcilar et al., 2009 [75]; Serdengecti et al., 2009 [82]; Cakir et al., 2009 [74]; Berker et al., 2010 [35]; Philip et al., 2014 [83]; Cansu et al., 2017 [84]; Faulkner et al., 2019 [25]

Studies	Patients (n)	Gender	Age	Thyroid Hemiagenesis
Veliz and Pineda, 2000	1	Female	35	Left
Hervas Benito et al., 2001	1	Male	45	Right
Zangeneh et al., 2001	1	Female	31	Left
Lee et al., 2003	1	Female	44	Left
Ozgen et al., 2004	1	Female	29	Left
Baldini et al., 2005	1	Female	41	Left
Ruchala et al., 2008	1	Female	49, 51	Left
Nebesio and Eugster, 2009	1	Female	10	Left
Kebapcilar et al., 2009	1	Female	43	Left
Serdengecti et al., 2009	1	Female	8	Left
Cakir et al., 2009	1	Female	55	Left
Berker et al., 2010	1	Male	63	Left
Philip et al., 2014	1	Female	50	Right
Cansu et al., 2017	1	Female	45	Left
Faulkner et al., 2019	1	Female	31	Left

TSH stimulation test and scintiscan with Tc-99 or Iodine had been used until the 1990s for differential diagnosis of GD and THA. Thyroid autoantibodies were used afterward and the TSH stimulation test was abandoned. Schechner et al. indicated thyroid-stimulating immunoglobulin (TSI) was more efficacious for distinguishing GD from toxic adenoma and the TSH stimulation test was obsolete in 1992 [76]. Thyrotropin receptor antibody (TRAb) measurement was first reported in a case report in 1994 [77].

Table 4 outlines a summary of case reports of THA with features of Hashimoto’s thyroiditis (HT) in which histology confirmation revealed chronic lymphocytic thyroiditis. A total of nine case reports were reviewed. All patients were females confirming the increased incidence in women with the majority of the THA occurring in the left lobe. A few of the cases had a conversion from HT to hyperthyroidism after treatment with thyroxine while another few were diagnosed with HT after initial treatment for hyperthyroidism with anti-thyroid medications. Finally, Table 5 highlights the congenital anomalies that have been documented with THA as a dysmorphic face with short stature and Down’s Syndrome.

**Table 4:** Summary of case reports of THA with Hashimoto’s thyroiditis THA: Thyroid hemiagenesis; ND: Not detected. Sharma et al., 2001 [85]; Ruchala et al., 2008 [70]; Nsame et al., 2013 [86]; Wang et al., 2017 [43]; Bosco et al., 2017 [87]

Study	Patients (n)	Gender	Age	Thyroid Hemiagenesis	Isthmus
Sharma et al., 2001	1	Female	33	Left	Present
Ruchala et al., 2008	1	Female	49	Left	ND
Nsame et al., 2013	1	Female	23	Right	Absent
Wang et al., 2017	1	Female	31	Left lobe and left PTG	ND
Bosco et al., 2017	1	Female	50	Left	Absent

**Table 5:** Summary of case reports of congenital anomalies with THA THA: Thyroid hemiagenesis; ND: Not detected. Vakili et al., 2003  [88]; Gursoy et al., 2006  [89]; Nebesio and Eugster, 2009  [73]; Ng et al., 2016  [90]; Ammar et al., 2016 [91]

Studies	Patients (n)	Gender	Age	Thyroid Hemiagenesis	Isthmus	Associated anomaly
Vakili et al., 2003	1	Female	14 months	Left	ND	Dysmorphic face with short statue
Gursoy et al., 2006	1	Male	19	Left	ND	Familial dilated cardiomyopathy and hypergonadotrophic hypogonadism
Nebesio and Eugster, 2009	1	Female	10	Left	Present	Down’s Syndrome
Ng et al., 2016	1	Male	37	Right	ND	Fourth brachial cyst
Ammar et al., 2016	1	Female	24	Right	ND	Brain Cavernoma and pituitary rathke cleft cyst

## Conclusions

THA is a rare congenital disorder affecting the thyroid gland. The awareness and understanding of this condition amongst doctors remain poor. Although there is a familial link, it largely remains sporadic. Females are reported to have a higher incidence of THA, like it is with other thyroid disorders. Most cases are diagnosed incidentally in euthyroid patients. THA may be associated with different pathologies including Graves’ disease, Hashimoto’s thyroiditis, etc. THA also adds to the cost and requirement for lifelong thyroid hormone replacement in those that have had thyroidectomies to remove a diseased thyroid tissue if required. Pre-operative diagnosis and awareness will certainly help healthcare workers and patients make informed decisions.
